# Hepatic Homeostasis of Metal Ions Following Acute Repeated Stress Exposure in Rats

**DOI:** 10.3390/antiox11010085

**Published:** 2021-12-29

**Authors:** Jereme G. Spiers, Li Si Tan, Stephen T. Anderson, Andrew F. Hill, Nickolas A. Lavidis, Hsiao-Jou Cortina Chen

**Affiliations:** 1 Department of Biochemistry and Genetics, La Trobe Institute for Molecular Science, La Trobe University, Bundoora 3083, Australia; andrew.hill@latrobe.edu.au; 2 School of Biomedical Sciences, The University of Queensland, St. Lucia, Brisbane 4072, Australia; tanlisi95@gmail.com (L.S.T.); stephen.anderson@uq.edu.au (S.T.A.); lavidis@uq.edu.au (N.A.L.); 3 Department of Pharmacology, Yong Loo Lin School of Medicine, National University of Singapore, Singapore 117600, Singapore; 4 Wellcome-MRC Institute of Metabolic Science-Metabolic Research Laboratories, University of Cambridge, Addenbrooke’s Hospital, Cambridge CB2 0QQ, UK

**Keywords:** copper, essential metals, iron, liver, rat, redox, stress, zinc

## Abstract

Essential metals such as copper, iron, and zinc are cofactors in various biological processes including oxygen utilisation, cell growth, and biomolecular synthesis. The homeostasis of these essential metals is carefully controlled through a system of protein transporters involved in the uptake, storage, and secretion. Some metal ions can be transformed by processes including reduction/oxidation (redox) reactions, and correspondingly, the breakdown of metal ion homeostasis can lead to formation of reactive oxygen and nitrogen species. We have previously demonstrated rapid biochemical responses to stress involving alterations in the redox state to generate free radicals and the resultant oxidative stress. However, the effects of stress on redox-active metals including iron and copper and redox-inert zinc have not been well characterised. Therefore, this study aims to examine the changes in these essential metals following exposure to short-term repeated stress, and to further elucidate the alterations in metal homeostasis through expression analysis of different metal transporters. Outbred male Wistar rats were exposed to unrestrained (control), 1 day, or 3 days of 6 h restraint stress (*n* = 8 per group). After the respective stress treatment, blood and liver samples were collected for the analysis of biometal concentrations and relative gene expression of metal transporter and binding proteins. Exposure to repeated restraint stress was highly effective in causing hepatic redox imbalance. Stress was also shown to induce hepatic metal redistribution, while modulating the mRNA levels of key metal transporters. Overall, this study is the first to characterise the gene expression profile of metal homeostasis following stress and provide insight into the changes occurring prior to the onset of chronic stress conditions.

## 1. Introduction

Transition metals including copper, iron, and zinc are key components for a variety of biological systems. Several of these essential trace metals are known to be redox-active, including copper and iron, making them catalytically active as cofactors in many metalloenzymes [1,2]. While zinc does not directly participate in redox reactions, it has the ability to modulate bound thiol groups in addition to acting as a stable biological cofactor required for the activity of more than 300 enzymes [3,4]. Thus, the metabolism and intracellular concentration of these metal ions is strictly regulated by metal transporters which mediate the import, distribution, and export from the cells to ensure normal enzymatic activity. For example, the uptake of copper into cells primarily occurs through copper transporter 1 (Ctr1) [5,6]. Upon entry, copper transfer is facilitated by specific copper chaperones such antioxidant 1 copper chaperone (Atox1) and copper chaperone for superoxide dismutase 1 (Ccs). Moreover, Atox1 shuttles copper to the secretory pathway, delivering it to copper transporting ATPases including both Atp7a and Atp7b within the trans-Golgi network [5,7]. The copper export protein, Atp7b, is almost exclusively expressed in the liver and incorporates copper into proteins such as apo-ceruloplasmin (Cp) for secretion and mediating the excretion of excess copper via bile [8]. In circulation, Cp is the major copper-binding protein, however, apart from distributing copper, secreted Cp plays an important role in iron metabolism by acting as a ferroxidase, mediating the oxidation of ferrous to ferric iron [9]. This catalytic oxidation allows the loading of ferric iron into transferrin and prevents the deleterious effects of Fenton chemistry where ferrous iron reacts with hydrogen peroxide to generate a hydroxyl radical [10]. Transferrin-captured ferric iron can then be transported to peripheral tissues and is recognised by transferrin receptor on cell membranes, followed by receptor-mediated endocytosis. A regulatory mechanism governing the expression of many iron metabolism-related genes including transferrin receptor involves the iron regulatory proteins 1 and 2 (IRPs; Irp1 and Irp2) [11,12]. This post-transcriptional control involves their binding to iron-responsive elements (IREs) in the untranslated regions of target mRNAs, thereby controlling mRNA translation and stability. Apart from transferrin receptor, another iron transporter also under the regulation of IRE-IRP system is ferroportin (Fpn1; also known as Slc40a1), the only mammalian iron exporter for non-heme iron known to date [13]. In response to infection, ferroportin is upregulated to mediate iron sequestration; however, in a state of iron deprivation, the expression is repressed by IRPs as well as several microRNAs [14,15,16].

We have previously demonstrated that short-term stress reduces cellular antioxidant defense capacity and induces hepatic oxidative and nitrosative stress [17]. This redox imbalance within the liver following stress is accompanied by an overall pro-inflammatory phenotype including upregulation of inducible nitric oxide synthase (iNOS) within hepatic macrophage/Kupffer cells [18]. Interestingly, it has been demonstrated previously that iNOS-derived nitric oxide plays a role in the regulation of intracellular zinc trafficking and homeostasis [19,20]. Specifically, pro-inflammatory cytokines including interleukin-1β, tumor necrosis factor, and interferon gamma increase iNOS-derived nitric oxide and induce cytoplasmic zinc release from the zinc storing protein, metallothionein [19]. Metallothionein also plays an important role in the cellular storage and/or delivery of copper ions to cuproenzymes, heavy metal detoxification, and cellular antioxidative defense [3,21]. In addition to metallothionein, zinc homeostasis is also governed by zinc transporters (ZnT), zinc importing proteins (Zrt and Irt-like protein; Zip), and metal response element (MRE)-binding transcription factor (Mtf) [22]. However, ferroportin has been shown to also accept zinc as a substrate and displays modest zinc transport activity during inflammation through the action of Mtf1 [23]. Specifically, Mtf1 is a key regulator of intracellular zinc that induces changes in the transcription of transporters such as ZnT1 and metallothionein in response to elevated zinc content and other stress stimuli such as oxidative stress and hypoxia [24,25,26]. Interestingly, Ohashi and colleagues [27] have demonstrated that following acute restraint stress in mice, zinc concentration in the liver was significantly elevated together with the zinc importer, Zip14, which may play a protective role against oxidative damage. While it is known that copper and iron-catalysed hydroxyl radicals are potent mediators of cellular oxidative stress, the contribution of stress on copper and iron homeostasis is less well characterised. Since the regulation of essential trace metals are tightly interlinked, this study aims to examine the changes in the three key essential metals, including copper, iron, and zinc, following exposure to short-term repeated stress, and to further elucidate the alterations in metal homeostasis through expression analysis of different metal transporters.

## 2. Materials and Methods

### 2.1. Experimental Animal

Eight-week-old outbred male Wistar rats (*Rattus norvegicus*; strain code: 003) weighing 298.30 ± 4.09 g were sourced from The University of Queensland Biological Resources and housed in a colony room within the Australian Institute of Biotechnology and Nanotechnology animal facility. Rats were housed in open-top cages under standard laboratory conditions (22 ± 2 °C; 55 ± 5% humidity) with an automatic 12 h light/12 h dark schedule (lights on at 07.00 h) and given ad libitum access to water and standard rat chow (Rat and Mouse Meat-Free Diet; Specialty Feeds, Glen Forrest, WA, Australia). This has a nutritional value of 14.2 MJ/Kg and is composed of 19% protein, 4.6% total fats, 59.9% total carbohydrates, and trace minerals including 70 mg/Kg iron, 16 mg/Kg copper, and 60 mg/Kg zinc. Rats were habituated to human handling for six consecutive days prior to any experimentation. Animals were treated in accordance with the Australian Code for the Care and Use of Animals for Scientific Purposes and all experimental procedures were approved by the University of Queensland Animal Ethics Committee under approval number SBS/456/14/URG.

### 2.2. Experimental Protocol

Rats were transported and acclimatised to the novel experimental room (within the same animal facility) on each experimental day 1 hour prior to any experimental procedures. Rats were randomly divided into four treatment groups of no stress (unstressed), acute (1 Day, single time of 6 h) and repeated (3 Days, 6 h/day) restraint stress from 9.00–15.00 h using adjustable wire mesh restrainers (*n* = 8 per group). Unstressed animals were deprived of food and water for the 6 h experimental period to isolate the treatment effect of restraint stress and were housed in a separate room within the same facility from animals exposed to restraint stress. At the end of each allocated treatment, rats were anesthetised with pentobarbital sodium (Lethabarb, 100 mg/kg, Virbac, Peakhurst, Australia; i.p.) and blood samples were collected via cardiac puncture into either ice-chilled tube with heparin (20 IU/mL blood) or plain tubes without the anticoagulant. Multiple liver punctures were then quickly removed using a biopsy punch and snap-frozen for storage at –80 °C for later relative mRNA/miRNA expression and biochemical analysis. Heparinised blood samples were centrifuged at 2000× *g* for 5 min and the supernatant plasma was collected and stored at −80 °C for later determination of Cp enzyme activity in addition to circulating copper and zinc concentrations. Blood samples collected in tubes without anticoagulant were left undisturbed at room temperature and allowed to clot for 15–30 min. Serum samples were collected following centrifugation at 2000× *g* for 10 min and stored at −80 °C for later determination of haptoglobin enzyme activity and ferric, ferrous, and total iron concentrations.

### 2.3. Haptoglobin Enzyme Activity Assay

The commercially available colorimetric Haptoglobin Kit (Phase™ Range TP801; Tridelta Development Ltd., Maynooth, Ireland) was used to measure haptoglobin concentration in plasma, based on its preservation of haemoglobin’s peroxidase activity. Following the manufacturer’s instructions, samples were loaded into a 96-well microplate and incubated with haemoglobin and chromogen. After 5 min, absorbance was measured at 620 nm with a 700 nm reference, using the Sunrise™ absorbance reader (Tecan Group Ltd., Männedorf, Switzerland). Sample haptoglobin was determined from a 0.039–2.5 mg/mL standard curve. Data were corrected for background and presented as mg/mL.

### 2.4. Ceruloplasmin Enzyme Activity Assay

The commercially available Ceruloplasmin Activity Colorimetric Kit (MAK177; Sigma-Aldrich, St. Louis, MO, USA) was used to measure Cp activity in the serum. Following the manufacturer’s instructions, chloride interference was removed using saturated ammonium sulfate (4.1 M; MAK177B; Sigma-Aldrich, St. Louis, MO, USA). Samples were briefly vortexed, incubated on ice for 5 min, and centrifuged at 10,000× *g* for 5 min. An aliquot of the resultant supernatant was dissolved in water at a ratio of 1:1, and loaded in duplicate to a 96-well microplate. A reaction mix was freshly made and added to each well using Cp assay buffer and Cp substrate. Absorbance was measured at 560 nm with a 700 nm reference, in kinetic mode for 15 min, using the Sunrise™ absorbance reader (Tecan Group Ltd., Männedorf, Switzerland). Sample Cp activity was determined from a 10–50 nmol/well standard curve of non-enzymatic oxidation. Data were corrected for background and presented as milli-unit/mL (mU/mL).

### 2.5. Liver Tissue Preparation

Liver tissues were homogenised with a T10 basic Ultra-Turrax^®^ rotor-stator homogeniser (IKA^®^ Works, Rawang, Malaysia) in 7.5 volumes (*w*/*v*) of 0.05 M Tris-HCl containing a protease inhibitor cocktail (P8340; Sigma-Aldrich), at top speed for 15–20 s. Aliquots of the whole homogenate were removed for zinc assay and protein determination. The remaining tissue homogenate was centrifuged at 10,000× *g* for 10 min at 4 °C and resultant supernatant collected for the determination of copper concentration. Hepatic iron level was determined from a separate fresh frozen liver punch.

### 2.6. Circulating and Liver Metal Concentrations

#### 2.6.1. Iron

A commercially available colorimetric Iron Assay Kit (MAK025; Sigma-Aldrich, St. Louis, MO, USA) was used to determine serum and hepatic iron concentrations according to the manufacturer’s instructions. Serum was used directly while liver tissue weighing approximately 30–50 mg were freshly-homogenised in 10 volumes (*w*/*v*) of iron assay buffer. Tissue homogenate was centrifuged at 13,000× *g* for 10 min, and resultant supernatant was collected for the assay. Serum or liver samples were loaded and an acidic assay buffer was added to all wells in a ratio of 1:1 (*v/v*), to separate iron from bound proteins and an iron reducer added for total iron measurement. Absorbance was measured at 593 nm with a 700 nm reference, using a Sunrise™ absorbance reader (Tecan Group Ltd., Männedorf, Switzerland). Data were corrected for background, normalised with corresponding protein content, and presented as μg/mL for serum iron and μg/mg for hepatic iron.

#### 2.6.2. Copper

A colorimetric Copper Assay Kit (MAK127; Sigma-Aldrich, St. Louis, MO, USA) was used to measure plasma and hepatic copper concentrations. According to manufacturer’s protocol, 100 μL of diluted plasma sample or supernatant from liver homogenate was added to 35 μL of assay reagent A, briefly vortexed, and centrifuged at 13,000× *g* for 2 min or 16,000× *g* for 20 min to pellet protein precipitates. The clear portion of resultant supernatant for both plasma and liver, were loaded into 96-well microplates in duplicates. A master reaction mix was made according to the protocol and added to each well. Following incubation, absorbance was measured using the Sunrise™ absorbance reader (Tecan Group Ltd., Männedorf, Switzerland) at 359 nm, with 650 nm reference. Plasma and hepatic copper concentrations were determined from respective standard curves with range of 4.68–300 μg/dL and 7.8–500 ng/dL. Data were corrected for background and corresponding protein concentration, and presented as μg/mL for plasma copper and μg/mg of protein for hepatic copper.

#### 2.6.3. Zinc

A colorimetric Zinc Assay Kit (MAK032; Sigma-Aldrich, St. Louis, MO, USA) was used to determine plasma and hepatic zinc concentrations. Following the manufacturer’s protocol, plasma and whole liver-homogenate samples were deproteinised with 7% trichloroacetic acid, briefly vortexed, and centrifuged at 13,000× *g* for 5 min. The resultant supernatants were loaded into 96-well microplates in duplicates. A zinc reagent mix was made according to the protocol and added to each well. Following incubation, absorbance was measured using the Sunrise™ absorbance reader (Tecan Group Ltd., Männedorf, Switzerland) at 560 nm, with 700 nm reference. Plasma and hepatic zinc concentrations were determined from a standard curve of 5–40 nmol/mL and 1.56–100 nmol/mL, respectively. Data were corrected for background and corresponding protein concentrations and presented as μg/μL for plasma zinc and ng/mg of protein for hepatic zinc.

### 2.7. mRNA Expression

Total RNA was isolated from liver tissues using the RNeasy mini kit (QIAGEN, Doncaster, Australia) and treated with deoxyribonuclease 1 (QIAGEN, Doncaster, Australia) according to the supplier’s protocol. Subsequently, 2 µg of RNA was reverse transcribed into cDNA using the iScriptTM reverse transcription supermix (Bio-Rad Laboratories, Gladesville, Australia). The cDNA was amplified using gene-specific TaqManTM Gene Expression Assays (Applied Biosystems, Foster City, CA, USA) and the assessed genes are listed in Table 1. Target gene expression was determined relative to a primer limited and well-characterised VIC-labelled beta-actin (ACTB; Applied Biosystems, Foster City, CA, USA) using the formula 2-ΔCt where ΔCt = (Ct target gene–Ct ACTB) and expressed relative to unstressed controls.

### 2.8. miRNA Expression

Small RNA was extracted using the miRNeasy Mini Kit (QIAGEN, Doncaster, Australia) and concentrations of total and small RNA were determined using the Agilent 2100 Bioanalyser with RNA 6000 Nano (5067–1511) and Small RNA (5067–1548) kits respectively (Agilent Technologies). A total of 50 ng of small RNA was reverse transcribed using a high-capacity cDNA reverse transcription kit (Applied Biosystems, Foster City, CA, USA) including pooled primers for the target miRNA of interest including miR-17 (Assay ID: 002308), miR-29a (Assay ID: 002112), and miR-485 (Assay ID: 462841). The miRNA expression was determined by Taqman gene expression ‘assay on demandTM’ assays relative to both snoRNA202 (Assay ID: 001232) and U6 (Assay ID: 001973) housekeeping miRNAs and subsequently expressed as fold change comparative to the unstressed control group using the 2–ΔCt method.

### 2.9. Protein Determination

Protein content was determined with a commercially available RED 660TM Protein Assay (G-Biosciences, St. Louis, MO, USA) using bovine serum albumin as a standard.

### 2.10. Data Analysis

All data in this study were analysed using GraphPad Prism 8.0.1 (GraphPad Software, Inc., San Diego, CA, USA). Data were first analysed for normality using the Brown–Forsythe test. One-way ANOVA with Fisher’s least significant difference (LSD) test was used to compare normally distributed data. Non-parametric Kruskal–Wallis ANOVA with Dunn’s multiple comparisons test were used for data sets with significantly different standard deviations. Unpaired *t*-tests were used to compare early miRNA expression in the liver between unstressed and Day 1 acute stress groups. Results were presented as mean ± standard error of the mean (±SEM) and p-values less than 0.05 were considered as statistically significant.

## 3. Results

### 3.1. Stress Increased Both Positive Acute Phase Proteins Including Haptoglobin and Ceruloplasmin Indicative of Liver Inflammation

**Figure 1:**
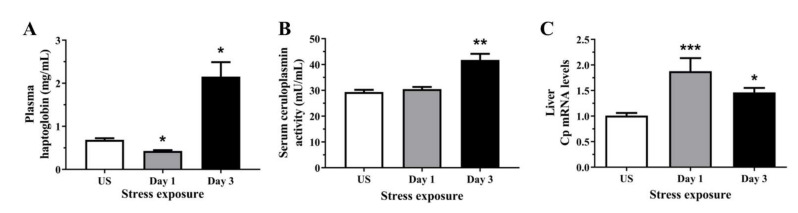
The effects of acute (Day 1, single 6 h) and repeated (Day 3, 6 h/day) restraint stress on (**A**) plasma haptoglobin, (**B**) serum ceruloplasmin activity, and (**C**) hepatic ceruloplasmin (Cp) mRNA expression compared to unstressed (US) rats (*n* = 6–8/group). Results were analysed using a non-parametric Kruskal–Wallis test with Dunn’s post-test. Data are expressed as mean ± SEM, * *p* < 0.05, ** *p* < 0.01, and *** *p* < 0.001.

Haptoglobin is a circulating acute phase protein produced by the liver and adipose tissue, induced by pro-oxidative conditions such as systemic inflammation. We observed a subtle decrease in plasma haptoglobin enzymatic activity following 1 day (*p* < 0.05) followed by an increase following 3 days (*p* < 0.05) of repeated restraint stress when compared to unstressed controls (Figure 1A). It is well known that as a positive acute phase reactant, Cp has ferroxidase activity which scavenges ferrous iron and free radicals. Figure 1B demonstrates that serum Cp activity was significantly elevated following 3 days (*p* < 0.01) of repeated restraint stress. As Cp is also synthesised in the liver, where it incorporates six copper atoms and is secreted into the circulation, we also looked at the mRNA expression of hepatic Cp. Similarly, we have observed a significant upregulation of liver Cp mRNA following 1 (*p* < 0.001) and 3 days (*p* < 0.01) of restraint stress (Figure 1C).

### 3.2. Acute Stress Decreases Plasma and Hepatic Copper Levels Demonstrating an Alteration in Copper Homeostasis

**Figure 2:**
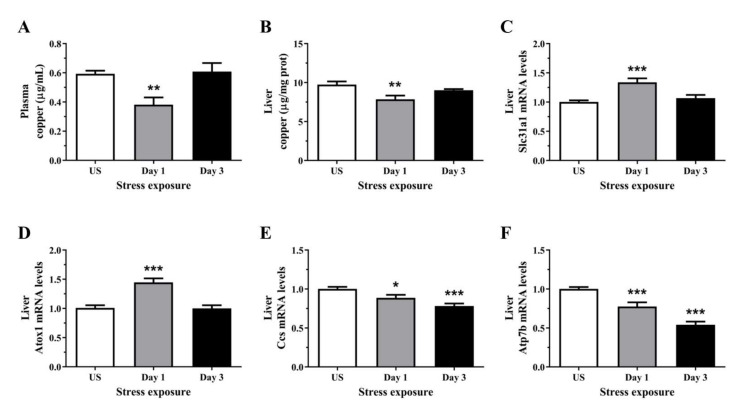
The effects of acute (Day 1, single 6 h) and repeated (Day 3, 6 h/day) restraint stress on (**A**) plasma copper, (**B**) liver copper, and hepatic (**C**) solute carrier family 31 member 1 (Slc31a1; Ctr1), (**D**) antioxidant 1 copper chaperone (Atox1), (**E**) copper chaperone for superoxide dismutase (Ccs), and (**F**) ATPase copper transporting beta (Atp7b) mRNA expression compared to unstressed (US) rats (*n* = 7–8/group). Results were analysed using one-way analysis of variance (ANOVA) with Fisher’s least significant difference (LSD) test. Data are expressed as mean ± SEM, * *p* < 0.05, ** *p* < 0.01, and *** *p* < 0.001.

As Cp is a major copper transport protein present in the plasma and is produced by the hepatic parenchymal cells, we next examined the levels of circulating and hepatic copper. Interestingly, plasma copper content was significantly decreased following day 1 (*p* < 0.01) of restraint stress, with no significant changes after repeated stress (Figure 2A). Similarly, post-test analysis showed hepatic copper concentration was decreased after day 1 (*p* < 0.01) of stress when compared to unstressed controls (Figure 2B). We then assessed the relative gene expression of key copper transporters in the liver. Figure 2C demonstrated significant increases in the mRNA levels of the high-affinity copper transporter, Ctr1, following 1 day (*p* < 0.001) of restraint stress. The subsequent delivery of imported copper to target cuproenzymes such as superoxide dismutase and cytochrome c oxidase relies on an elegant metallochaperone system. Several cytoplasmic chaperones have been described including Atox1 (copper metallochaperone) and Ccs (copper chaperone for Cu-Zn superoxide dismutase). Stress significantly increased hepatic Atox1 expression following 1 day (*p* < 0.001) of restraint stress (Figure 2D). In contrast to Atox1, the second copper chaperone, Ccs, is a protein required for the delivery of copper to copper-zinc superoxide dismutase. Figure 2E revealed that stress progressively decrease Ccs mRNA levels after day 1 (*p* < 0.05) and 3 (*p* < 0.001) of restraint stress when compared to unstressed controls. Copper delivery to the secretory pathway is further facilitated by the membrane-associated copper-transporting ATPases including Atp7b. We have demonstrated in Figure 2F that stress induces a sustained suppression of Atp7b mRNA levels at both time-points examined (*p* < 0.001).

### 3.3. Zinc Content Was Reduced in the Plasma but Increase in the Liver Following Repeated Acute Stress

**Figure 3:**
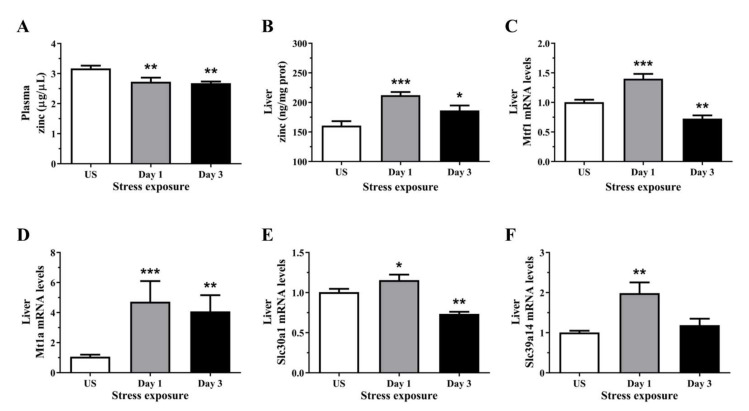
The effects of acute (Day 1, single 6 h) and repeated (Day 3, 6 h/day) restraint stress on (**A**) plasma zinc, (**B**) liver zinc, and hepatic (**C**) metal regulatory transcription factor 1 (Mtf1), (**D**) metallothionein 1A (Mt1a), (**E**) solute carrier family 30 member 1 (Slc30a1; Znt1), and (**F**) solute carrier family 39 member 14 (Slc39a1; Zip14) mRNA expression compared to unstressed (US) rats (*n* = 7–8/group). Results in (**A**–**E**) were analysed using one-way ANOVA with Fisher’s LSD test; (**D**,**F**) were analysed using non-parametric Kruskal-Wallis test with Dunn’s post-test. Data are expressed as mean ± SEM, * *p* < 0.05, ** *p* < 0.01, and *** *p* < 0.001.

Repeated restraint stress significantly reduced plasma zinc concentrations after 1 and 3 episodes (*p* < 0.01) of restraint stress (Figure 3A). Conversely, hepatic zinc concentration was significantly increased after 1 (*p* < 0.001) and 3 days (*p* < 0.05) of restraint stress, relative to controls (Figure 3B). To further demonstrate the effects of stress on zinc homeostasis, we assessed the changes in the relative gene expression of hepatic zinc sensor which coordinates the expression of genes involved in zinc homeostasis. We found that stress significantly increased the expression of the cellular zinc sensor Mtf1 following 1 day (*p* < 0.001) of acute stress, but was downregulated after 3 (*p* < 0.05) episodes of repeated stress (Figure 3C). Only a small number of genes are known direct targets of the zinc-responsive transcription factor Mtf1, these includes metallothionein and zinc-transporter-1 (Znt1). Figure 3D demonstrates a strong upregulation in Mt1a mRNA following 1 (*p* < 0.001) and 3 days (*p* < 0.01) of restraint stress. The expression of Znt1 was significantly increased following 1 day (*p* < 0.05) of 6 h restraint stress and was reduced following 3 days (*p* < 0.01) of repeated stress exposure (Figure 3E). In addition to the Znt family, we also examined the family of zinc transporters including the Zrt-, Irt-like protein (Zip; Slc39). Hepatic Zip14 mRNA level was significantly increased after 1 day (*p* < 0.01) of restraint stress when compared to unstressed controls (Figure 3F).

### 3.4. Acute Stress Leads to an Overall Reduction in Serum and Hepatic Liver Iron Content

**Figure 4:**
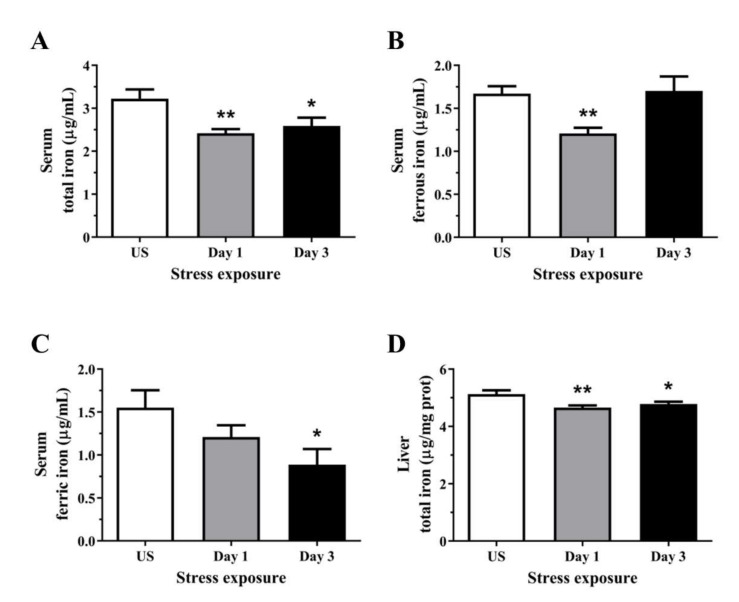
The effects of acute (Day 1, single 6 h) and repeated (Day 3, 6 h/day) restraint stress on (**A**) serum total iron, (**B**) serum ferrous iron, (**C**) serum ferric iron, and (**D**) liver total iron levels compared to unstressed (US) rats (*n* = 6–8/group). Results were analysed using one-way ANOVA with Fisher’s LSD test. Data are expressed as mean ± SEM, * *p* < 0.05, and ** *p* < 0.01.

The total iron concentration in the serum was decreased following day 1 (*p* < 0.01) and 3 (*p* < 0.05) of restraint stress (Figure 4A). Cellular iron usually exists in the ferrous form, and Figure 4B shows transient reductions in circulating ferrous iron following a single episode of stress (*p* < 0.01), returning to similar levels as unstressed controls following 3 days of repeated stress. Interestingly, the levels of iron in the ferric state were decreased following 3 days (*p* < 0.05) of repeated stress treatment (Figure 4C). One important function of the liver is the regulation of iron homeostasis and in the present study, 1 (*p* < 0.01) and 3 episodes (*p* < 0.05) of 6 h acute stress decreased hepatic total iron content (Figure 4D).

### 3.5. The Decrease in Serum and Hepatic Iron Content and Upregulation of Iron Transporters Suggests There Is a Redistribution of Iron in Response to Stress

**Figure 5:**
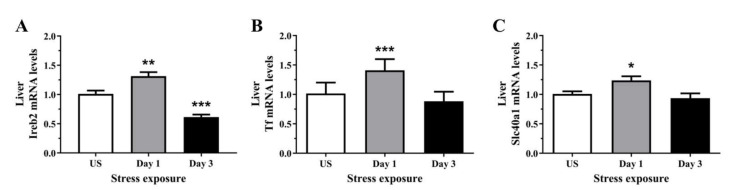
The effects of acute (Day 1, single 6 h) and repeated (Day 3, 6 h/day) restraint stress on hepatic (**A**) iron responsive element binding protein 2 (Ireb2; Irp2), (**B**) transferrin (Tf), and (**C**) solute carrier family 40 member 1 (Slc40a1; Fnp1) mRNA expression compared to unstressed (US) rats (*n* = 8/group). Results were analysed using one-way ANOVA with Fisher’s LSD test. Data are expressed as mean ± SEM, * *p* < 0.05, ** *p* < 0.01, and *** *p* < 0.001.

**Figure 6:**
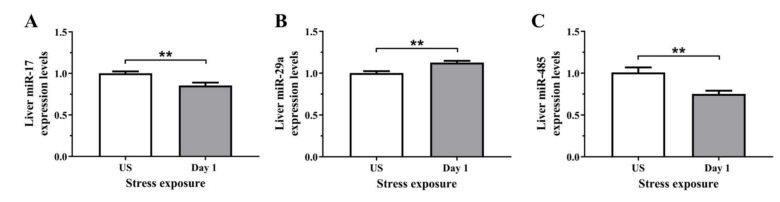
The effects of acute (Day 1, single 6 h) restraint stress on hepatic (**A**) miR-17, (**B**) miR-485, and (**C**) miR-29a miRNA expression compared to unstressed (US) rats (*n* = 6/group). Results were analysed using unpaired *t*-test. Data are expressed as mean ± SEM, ** *p* < 0.01.

To determine if the stress-induced changes in serum and hepatic iron content were preceded by alterations at the molecular level, we assessed the relative gene expression of proteins involved in iron regulation, transport, and binding, in the liver. The regulator of cellular iron homeostasis, Ireb2 (Irp2), showed a significant upregulation after 1 episode of restraint stress (*p* < 0.01), and was downregulated after 3 days (*p* < 0.001) of stress (Figure 5A). Furthermore, the mRNA levels of transferrin were increased following 1 day (*p* < 0.001) of stress exposure when compared to unstressed controls (Figure 5B). Similarly, Figure 5C further demonstrates that the iron exporter, Fpn1, was significantly up-regulated following 1 day (*p* < 0.05) of acute restraint stress in the liver (Figure 5C). Next, as most of the changes in iron regulation was observed following one day of stress exposure, we examined three microRNAs that have previously demonstrated regulatory roles in iron homeostasis. Figure 6A,B revealed that liver miR-17 and miR-485 were both significantly downregulated following 1 episode (*p* < 0.01) of 6 h restraint stress. Furthermore, another potential upstream driver of iron-responsive gene expression, miR-29a, was upregulated in the liver following 1 day (*p* < 0.01) of stress exposure (Figure 6C).

## 4. Discussion

The present study has shown that acute repeated stress induces dynamic changes in key metal transporter expression and hepatic metal distribution of copper, iron, and zinc. Stress can shift the redox balance of several tissue types, including erythrocytes, liver, and specific neural regions towards an increasingly oxidative state in both acute and chronic stress profiles [17,28,29,30]. As metal-catalysed hydroxyl radicals are potent mediators of cellular oxidative injury, the observed changes in metal homeostasis following stress in the liver may be a significant contributing factor to the hepatic redox imbalance observed in our previous work. The changes in regulatory miRNA and mRNA of key metal transporters highlight that acute stress induces rapid changes at a network level to influence biometal distribution in the liver and throughout the body via serum carriers.

Following acute repeated stress, we observed an increase in plasma haptoglobin which is predominately produced in the liver and protects against potential threats of an acute phase reaction such as stress and inflammation [31]. Studies have demonstrated hepatic expression and circulating levels of haptoglobin synergistically increase with both endogenous and synthetic glucocorticoids [32,33,34]. Previous studies have also demonstrated that increased haptoglobin levels and oxidative stress can be induced by post-weaning stress and 7-day heat stress in piglets [35,36]. In response to redox imbalance, haptoglobin can also function as an antioxidant involved in preventing haemoglobin-driven generation of hydroxyl radicals and lipid peroxides by stabilising ferric irons [37,38]. Haptoglobin has also been shown to scavenge nitric oxide through rapid and irreversible binding, thereby limiting its bioavailability and preventing nitrosative damage from reactive nitrogen species [39]. Another acute-phase protein, Cp, is also produced primarily by the liver and increases during inflammation [40,41]. Similar to the observed increase in haptoglobin, Cp activity in the serum was increased following 3 days of repeated stress with an accompanying increase in Cp mRNA at both stress time-points examined. In addition to being the major copper transport protein in circulation, Cp is also a multifunctional enzyme exhibiting nitric oxide oxidase, nitrite synthase, superoxide dismutase, and most importantly, ferroxidase activities [42,43]. The oxidation of ferrous to ferric iron exemplifies the important antioxidant property of Cp as the resultant ferric iron binds to transferrin, preventing reactions of Fenton chemistry that generate free radicals. Moreover, the essential role of Cp in iron metabolism is particularly evident in the case of loss-of-function mutations in the Cp gene, resulting in systemic iron overload with clinical features including diabetes, liver disease, and neurodegeneration, collectively known as aceruloplasminaemia [44]. Plasma Cp binds up to 95% of total circulating copper and, although copper does not affect the rate of Cp synthesis or secretion, Cp secreted without copper incorporation results in an unstable apoprotein devoid of oxidase activity that is rapidly degraded in circulation [45,46,47].

The observed changes in Cp led us to examine copper homeostasis in stressed rats. We observed copper concentrations in both plasma and liver significantly decreased after 6 h of stress exposure. This response could be indicative of copper sequestration by other organs during acute stress. One possible candidate is the brain, which concentrates copper for metabolic functions and antioxidant defence, and has previously demonstrated alterations in copper levels following acute stress [48,49]. Upon repeated exposures to restraint stress, the decrease in plasma and liver copper levels recovered, suggesting restoration of copper homeostasis during repeated stress exposure. In support of this, we demonstrated mRNA expression of the high affinity influx copper transporter (Ctr1) and copper metallochaperone (Atox1) were significantly upregulated following stress exposure. The observed increase in Ctr1 of approximately 30% following acute stress likely facilitates the replenishment of liver copper levels. Following the import of copper ions into hepatocytes from plasma proteins via Ctr1 and potentially other yet unidentified transporters, previous work has shown copper is carried via the chaperone Atox1 to the ATP-dependent pump, Atp7b, as part of the secretory pathway in the trans-Golgi network [50,51]. In the lumen of the trans-Golgi network, copper is then incorporated into apoceruloplasmin and this holoprotein is released into the blood plasma via exocytosis. In response to copper stress within hepatocytes, Atp7b can translocate to the biliary pole and transport excess copper out of the cell for subsequent copper excretion via the bile [52]. However, the rapid downregulation in hepatic Atp7b following stress observed in this study could explain the observed decrease in plasma copper following 1 day of stress exposure. In addition to the Atox1-Atp7b copper trafficking route, intracellular copper is also integrated into the other major copper chaperone, Ccs, which is responsible for copper incorporation to the antioxidant copper-zinc superoxide dismutase (Sod1) for its catalytic activity. The activity of Sod1 can be affected by alterations in copper homeostasis, with liver-specific deletion of Ctr1 resulting in a 42% decrease in Sod1 activity [53]. Interestingly, despite the observed downregulation in Ccs expression, we have previously demonstrated a rapid upregulation in Sod1 mRNA in the liver following acute stress with a corresponding increase in protein expression following 3 days of repeated restraint stress [17]. Copper incorporation into Sod1 can also be Ccs-independent, instead utilising reduced glutathione [54]. Together, this highlights a potential mechanism through which acute stress can induce subtle alterations in copper homeostasis to shift cellular redox balance, via limiting the available biometal for Sod1 holoenzyme formation.

The present study also examined the effect of stress on antioxidant zinc homeostasis. Overall, circulating zinc concentrations were reduced whilst liver zinc was significantly higher in all stress groups compared to unstressed controls. These results agree with previous studies which employed both acute and chronic stress models in addition to in vitro studies using primary hepatocytes treated with synthetic glucocorticoids [27,55,56,57]. Furthermore, zinc deficiency has been observed in individuals suffering from anxiety and depression, whilst a zinc-deficient diet has been shown to influence the severity of depressive symptoms in women but not men [58,59,60]. We also examined the mRNA expression of major hepatic zinc-binding and transport proteins including Mtf1, Mt1a, Slc30a1 (ZnT1), and Slc39a14 (Zip14). After a single episode of 6 h restraint stress, all four of these genes were significantly upregulated. As a cellular zinc sensor, Mtf1 is known to regulate the transcription of zinc transporters including metallothioneins and ZnT1 [61]. In response to the transcriptional control by Mtf1, the consistent upregulation of hepatic Mt1a gene expression observed in the present study concurs with previous reports showing metallothionein induction by restraint and immobilisation stress [62,63]. This may be a protective mechanism as cysteine-rich metallothionein is involved in the reduction of hydroxyl radicals produced under stressful conditions [64]. Zinc can also exert antioxidant activity by acting as an inhibitor of NADPH oxidase which results in decreased generation of superoxide free radical [65]. Interestingly, the zinc exporter ZnT1 showed an initial increase in mRNA levels before becoming significantly downregulated following repeated stress exposure which could facilitate hepatic zinc sequestration in these longer stress durations. Zinc accumulation by the liver is also supported by the significant increase in the zinc importer, Zip14, which is comparable with previous studies [27,56]. Furthermore, Liuzzi and colleagues [66] have shown that hepatic Zip14 is highly responsive to inflammatory cytokines including interleukin-6 and is responsible for producing the hypozincemia in circulation observed in an acute phase response. In a metabolic stress model, Zip14-mediated hepatic zinc uptake plays an important role in suppressing endoplasmic reticulum stress-induced apoptosis and hepatic steatosis [67]. Together, this highlights how sensitive zinc homeostasis is to acute perturbations in stress, dynamically regulating the availability and storage of this biometal in redox-sensitive tissues such as the liver.

In addition to the hypozincemia, the acute phase response is often accompanied by hypoferremia. Comparably, we have observed a significant reduction in total iron content in the serum which is in agreement with previous studies demonstrating that a 3-day psychological stress paradigm decreases serum iron levels and inhibits erythropoiesis [68]. We further examined the effects of acute repeated stress on the oxidation states of iron which showed a rapid decrease and subsequent recovery in the labile and catalytically active ferrous form. Conversely, although we have shown an increase in serum Cp activity following repeated stress exposure, the levels of ferric iron were reduced at both of these time-points. This is intriguing, as Cp oxidises ferrous to ferric iron to enable cellular iron transport via transferrin binding. However, as the activity of Cp depends greatly on the availability of copper and we observed a decrease in plasma and liver copper following stress, this may suggest the catalytic activity of Cp has been compromised. The total iron level in the liver was subtly decreased following a 6 h acute restraint stress with subsequent recovery, indicating the liver regulates iron concentrations in a robust and rapid manner. In addition to measuring the circulating and liver iron concentrations, we analysed the relative gene expression of key iron-binding and transport proteins in the liver. Intracellular iron homeostasis in mammals is maintained by Ireb2, one of the two ubiquitously expressed iron-responsive element-binding proteins (IRPs). Stress exposure induced an initial increase in Ireb2 mRNA expression followed by significant reductions with repeated exposures to restraint. Furthermore, acute stress increased transferrin mRNA which aligns with the decrease in liver total iron observed following 6 h of restraint stress. Guo and colleagues [69] have further demonstrated that prolonged restraint stress exposure for 14 consecutive days induces liver iron accumulation in addition to downregulating transferrin expression. Following one episode of restraint stress, the sole known cellular exporter of elemental iron, ferroportin (Slc40a1), was upregulated. Apart from the conventional regulation by IRPs, the increase in ferroportin could be due to a suppression in miR-17 as described in a previous study by Kong and colleagues [15]. Overexpression of miR-485, another microRNA known to regulate ferroportin post-transcriptionally, can repress ferroportin expression which leads to increased cellular iron. Conversely, inhibition of miR-485-3p activity via mutation of its target sites on ferroportin mRNA reverses the repression resulting in decreased cellular iron levels [14]. In addition to targeting Ireb2, miR-29a promotes pro-inflammatory processes known to alter metalloprotein expression [70]. Our previous work has demonstrated stress exposure promotes polarisation of the hepatic Kupffer cells, inducing hepatic inflammation and oxidative stress [18]. This further resulted in a downregulated Nrf2-mediated antioxidant pathway, reduced cysteine thiol content, protein carbonyl formation and lipid peroxidation, all key contributors to ferroptosis [17]. Despite these changes, glutathione peroxidase 4 expression was transiently increased following restraint stress, suggesting there may be a degree of hepatic protection from ferroptosis-mediated cell death despite the accumulation of other oxidative stress markers. In addition to this, the reduction in iron observed in this present study suggest the liver may also transiently reduce hepatocellular iron content to selectively prevent ferroptosis during periods of repeated stress. Taken together, in response to the stress-induced reduction of liver iron, both miRNA regulation and binding of Ireb2 to the iron-responsive element in the untranslated regions of ferroportin mRNA may inhibit its translation, allowing the liver to reduce iron export and prevent further loss of cellular iron [11].

## 5. Conclusions

In summary, the liver plays a central role in regulating iron, copper, and zinc homeostasis, both within hepatic tissues and via production of circulating plasma/serum binding proteins responsible for wider distribution throughout the body. Despite their critical importance, these essential metals can also impede normal physiological functions when their levels, distribution, and redox state are altered. Specifically, we have identified restraint stress increases the key biometal regulatory acute phase proteins haptoglobin and ceruloplasmin which was accompanied by reductions in copper, zinc, and iron in circulation. Stress exposure further disrupted biometal homeostasis in the liver, reducing copper and iron whilst increasing zinc tissue concentrations. This was accompanied by changes in the hepatic expression of key transporters and chaperones for copper (Slc31a1, Atox1, Ccs, and Atp7b), zinc (Mtf1, Mt1a, Slc30a1, and Slc39a14), and iron (Ireb2, Tf, and Slc40a1). Finally, we showed changes in regulatory miRNA known to target ferroportin (miR-17 and miR-485) and Ireb2 (miR-29a) which may also have wider implications on hepatic inflammation and oxidative stress during stress. The sensitivity of the hepatic biometal network to acute restraint highlights the far-reaching implications of repeated psychological stressors and may begin to explain how stress can induce profound changes in oxidative stress throughout the body.

## Figures and Tables

**Table 1:** List of TaqmanTM gene expression assays used for quantification of mRNA levels.

	Gene Name	Gene Symbol	TaqMan Assay ID
**Copper**	Ceruloplasmin	Cp	Rn00561049_m1
	Copper transporter 1 (Ctr1)	Slc31a1	Rn00683634_m1
	Antioxidant 1 copper chaperone	Atox1	Rn00584459_m1
	Copper transporting ATPase 7 beta-polypeptide	Atp7b	Rn00560862_m1
	Copper chaperone for SOD	Ccs	Rn00584772_m1
**Zinc**	Metallothionein 1a	Mt1a	Rn00821759_g1
	Zrt- and Irt-like protein 14 (Zip14)	Slc39a14	Rn01468336_m1
	Zinc transporter 1 (Znt1)	Slc30a1	Rn00575737_m1
	Metal-regulatory transcription factor 1	Mtf1	Rn01749440_m1
**Iron**	Iron responsive element binding protein 2 (Irp2)	Ireb2	Rn00575852_m1
	Transferrin	Tf	Rn01445482_m1
	Ferroportin (Fpn1)	Slc40a1	Rn00591187_m1

## Data Availability

Data is contained within the article.
